# ABO Incompatibility and Hematopoietic Stem Cell Transplantation Outcomes

**Published:** 2017-04-01

**Authors:** Mohammad Vaezi, Davoud Oulad Dameshghi, Maryam Souri, Seyed Amin Setarehdan, Kamran Alimoghaddam, Ardeshir Ghavamzadeh

**Affiliations:** 1 Hematology- Oncology and Stem Cell Transplantation Research Center, Tehran University of Medical Sciences, Tehran, Iran; 2 Qom University of Medical Sciences, Qom, Iran

**Keywords:** ABO incompatibility, Allogeneic hematopoietic stem cell transplantation

## Abstract

**Introduction:** The increased risk of hemolytic reactions and erythrocyte recovery delay in ABO incompatible hematopoietic stem cell transplantation (HSCT) are well established. Effects of ABO incompatibility on other transplantation outcomes are evaluated in this study.

**Subjects and Methods:** We prospectively followed 501 patients undergoing allogeneic stem cell transplantation regarding their ABO compatibility groups for a median time of 34.7 months. Patients were studied in minor, major and bidirectional mismatched and matched groups.

**Results: **Mean survival time (OS) was lower in minor mismatched group (p-value= 0.017). Minor and bidirectional mismatched groups received significantly more packed cell units than matched group (p-value < 0.0001 and p-value =0.002, respectively).Mean number of platelet unit infusion was significantly more in major mismatched recipients than matched group (p- value=0.031). Death rate was much more than expected in minor mismatched group. Two cases of PRCA (pure red cell aplasia) were found in major mismatched group. No statistically significant difference was found in the incidence of acute GVHD, chronic GVHD, time to neutrophil recovery, relapse- free survival, non-relapse mortality and relapse rate among groups.

**Conclusion: **In order to prevent complications of ABO-incompatible SCT such as decrease in OS and the need for more transfusions, choosing ABO-compatible donors would improve transplantation outcomes.

## Introduction

 In contrast to solid organ transplantation, HLA matching is critical in allogeneic hematopoietic stem cell transplantation (HSCT) and ABO incompatibility is not considered a barrier, but it seems that erythrocyte recovery delay and hemolytic complications maybe a problem by the presence of recipient antibodies against donor ABO antigens. The increased risk of hemolytic reactions in ABO incompatible HSCT is well understood.^1^Theeffect of ABO incompatibility on transplantation such as overall survival (OS), graft-versus-host disease (GVHD), and relapse is still controversial.^2^^,^^8^ ABO incompatibility has three features: major incompatibility, that happens when the recipient with O blood group receives graft from A/B/AB donor, minor incompatibility which occurs when the donor with anti A/B antibodies donates stem cells to a patient with A/B or AB blood group and bidirectional incompatibility is defined when both donor and recipient have anti ABO antibodies. In this study, we evaluated graft outcomes regarding ABO incompatibility which has been a conflicting issue during recent years. If ABO incompatibility deteriorates graft outcomes, choosing the better donor may improve HSCT results, albeit if possible.

## SUBJECTS AND METHODS

 We prospectively followed the patients (n=501) undergoing allogeneic stem cell transplantation between 2010 and 2012 in our center for a median time of 34.7 months. Patients’ characteristics are shown in Table1.

**Table1:** Patients’ characteristics

		Total	Match	Major mismatch	Minor mismatch	Bidirec- tional	P value
Recipient age							
	Mean (Median)	24.3 (23)	24.8 (25)	22.9 (19)	25.9 (23)	19.8 (16)	0.034
	Range	1-63	1-57	1-63	1-61	1-54	
Recipient gender							
	Male	298	174	55	53	16	0.32
	Female	203	117	46	26	14	
Gender mismatch							
	D-R Sex-Match	245	144	47	40	14	0.422
	Female to Male	138	73	31	27	7	
	Male to Female	118	74	23	12	9	
Conditioning regimen							
	Myeloablative	442	252	92	70	28	0.512
	Non-myeloablative	59	39	9	9	2	
ATG in Conditioning							
	Yes	115	52	32	23	8	0.013
	No	385	239	69	55	22	
HLA type							
	Full match sibling	475	279	93	76	27	0.23
	HLA matched other relative	16	6	5	2	3	
	HLA mismatch relative	10	6	3	1	0	
Primary diseases							
	Malignant	318	192	56	52	18	0.268
	Benign	183	99	45	27	12	
Stem cell source							
	PBSC	456	270	86	72	28	0.223
	BM	38	16	14	6	2	
	CB	7	5	1	1	0	
Mononuclear Cell Dose *108/Kg	Mean (Median)	8.02 (8.04)	8.35 (8.04)	7.20 (8.03)	8.05 (8.07)	7.44 (8.02)	0.26
CD34 cell dose*106/Kg	Mean (Median)	4.03 (3.7)	4.06 (3.85)	4.01 (3.25)	3.93 (3.5)	4.04 (3.77)	0.68
CD3 cell dose*106/Kg	Mean (Median)	274.04 (284)	275.52 (289.5)	281.91 (288)	262.79 (274)	262.99 (260)	0.36

PBSC: peripheral Blood Stem Cell, BM: Bone Marrow, CB: Cord Blood,

Malignant diseases (n=318) included acute myeloid leukemia (AML), acute lymphoid leukemia (ALL), chronic myeloid leukemia (CML), myelodysplastic syndrome (MDS), malignant lymphoma and multiple myeloma (MM), while severe aplastic anemia (SAA), thalassemia, Fanconi anemia, osteopetrosis and leukocyteadhesion deficiency syndrome were defined as benign disorders (n=183). Four hundred forty-two patients received myeloablative (MA) conditioning regimen and others (n=59) were transplanted with non-myeloablative regimen.

Stem cell source was peripheral blood in 456 patients and the rest received cells from bone marrow (n=38) and cord blood (n=7). Acute GVHD (aGVHD) grade was determined according to Glucksberg system by presentation and staging of gastrointestinal, liver and skin GVHD at least seven days after transplantation.^9^Absolute neutrophil count (ANC) more than 0.5 *109/L for 3 consequent days was considered as neutrophil recovery and platelet engraftment was determined by platelet count of greater than 20*109/L for three consequent days without any supplementary platelet. Chimerism was assessed on days +15, +30, +60 and +90. Relapse and secondary graft failure were identified by clinical and /or hematologic recurrence or chimerism decline. Death due to treatment except relapse was defined as none-relapse mortality (NRM). Dates of relapse and death were recorded to identify relapse-free survival (RFS) and overall survival (OS). All participants signed the informed consent forms.

**Statistical Analysis**

Statistical analysis was performed using SPSS version 22.0. The incidences of death, relapse, acute and chronic GVHD were compared in each ABO blood group incompatibility using cross-tab tables with likelihood-ratio χ2 statistics. The means of recipient’s and donor’s age, platelet and WBC engraftment, packed cell and platelet infusion were compared using ANOVA and Kruskal-wallis with post-hoc statistics. Overall survival and relapse-free survival were analyzed by the Kaplan-Meier method, and the Breslow test was used to examinesignificant differences among blood group compatibilities. Factors that significantly affected survival and relapse-free survival were evaluated by the Cox proportional hazards multivariate model. Logistic regression multivariate analysis was used to determine significant effects of variables on incidence of mortality, relapse, and non-relapse mortality, acute and chronic GVHD as outcomes.^10^^, ^^12^

## Results

 According to ABO compatibility of donors and recipients, four groups were distinguished: match, major mismatch, minor mismatch and bidirectional ABO mismatch. Recipient’s gender, gender mismatch, conditioning regimen, HLA matching, primary disease, stem cell source, receiving ATG(anti-thymocyte globulin) in conditioning regimen, mononuclear cells, CD34 and CD3 cell doses were almost equally distributed in these four groups. Only recipient’s age was different among groups (p-value= 0.034). Table 1 shows univariate analysis of patients’ characteristics.

Univariate analysis of transplantation outcomes regarding BO compatibility groups showed no significant difference inaGVHD, cGVHD, time to neutrophil recovery, NRM and relapse rate in all groups. Chimerism was different among groups, but it was not significant (p-value=0.078).Mean days of platelet engraftment, units of packed cell transfusion, units of platelet infusion and death incidence rate were statistically different (Table 2).

Minor and bidirectional mismatched groups received significantly more packed cells than matched group (p- value<0.0001 and p-value =0.002, respectively). Although major mismatched patients received more packed cells than matched group, the difference was not significant (p value =0.06).The Mean number of platelet units transfused was significantly more in major mismatched recipients than matched group(p-value=0.031). Death rate was less than expected in matched group and it was much more than expected in minor mismatched group(Table2).

Totally, overall survival was 39.8 months (95% CI: 38.1 – 41.5). In univariate analysis, mean survival time (OS) was statistically different among groups (p value= 0.017) and the worst result was found in minor mismatched one .Relapse-free survival (RFS) was 45.3 (95% CI: 44.0 – 46.7) in all patients and it was lower in minor mismatched group, but the difference was not statistically significant (p value=0.30) 

In multivariate analysis of overall survival, minor mismatch ABO incompatibility decreased survival (RR: 2.29, CI: 1.47-3.55, p value<0.0001). Other ABO incompatibilities did not affect survival (Table 3, Figure1-A).Malignant diseases decreased OS (RR:2.62,CI:1.66-4.14,p-value<0.0001).Grade II to IV acute GVHD, both GI and liver deteriorated the outcome(RR:1.80,CI:1.26-2.57,p-value=0.001 and RR:2.70,CI:1.18-6.17,p-value=0.018,respectively),while limited cGVHD improved OS(RR: 0.48,CI: 0.29-0.81, p value=0.006)(Table3).

**Table 2:** Post- transplantation outcomes by ABO incompatibility

		Total	Match	Major mismatch	Minor mismatch		Bidirec- tional		P value	
AGVHD Grade ≥ II										
	Yes	212	112	47		37		16		0.190
	No	274	171	49		40		14		
CGVHD										
	Yes	189	105	37		32		15		0.46
	No	312	186	64		47		15		
Chimerism										
	> 95%	442	257	92		64		29		0.078
	< 95%	59	34	9		15		1		
Neutrophil recovery (+Days)										
	Mean (Median)	14.02 (13)	13.59 (13)	14.98 (13)		13.95 (13)		15.23 (13)		0.64
	Range	1-66	3-27	9-66		8-44		1-51		
Plt engraftment (+Days)										
	Mean (Median)	17.97 (16)	17.15 (15)	19.78 (17)		18.66 (16)		18.07 (15)		0.04
	Range	4-101	4-101	10-71		8-69		10-38		
Packed Cell infusion (Unit)	Mean (Median)	2.48 (1)	1.83 (1)	2.89 (1)		3.85 (3)		3.80 (4)		<0.0001
Platelet infusion (Unit)	Mean (Median)	4.42 (2)	3.51 (2)	6.18 (2)		5.18 (2)		5.43 (3)		0.002
Relapse^1^										
	Yes	75	44	12		16		3		0.38
	No	426	247	89		63		27		
Death^1^										
	Yes	139	68	30		31		10		0.034
`	No	362	223	71		48		20		
Non-Relapse Mortality										
	NRM	73	32	18		15		8		0.193
	RM	66	36	12		16		2		

1 Medianfollow-up: 34.72 months (33.26 – 36.18)

**Figure1-A:**
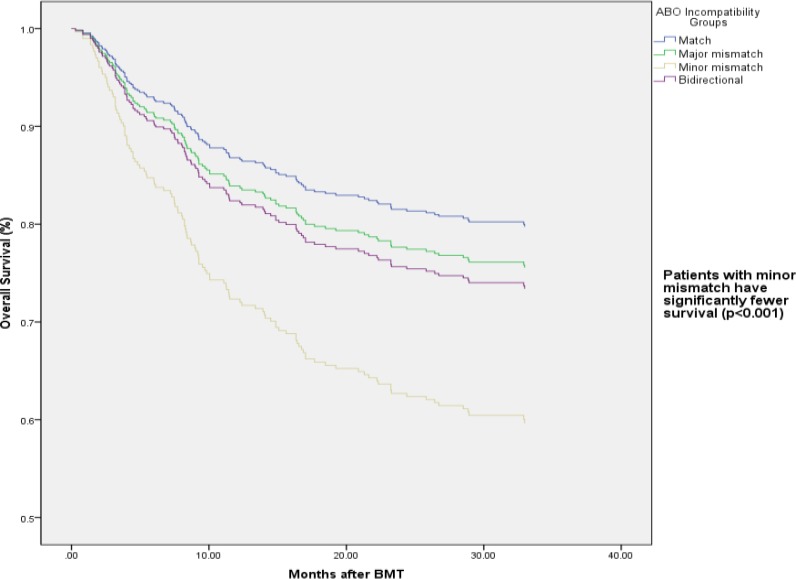
Overall Survival by ABO incompatibility Groups in multivariate Cox regression analysis

**Table 3:** Multivariate analysis for overall survival and relapse-free survival

Factors	Overall Survival		Relapse-Free Survival	
	Relative Risk (95% CI)	P value	Relative Risk (95% CI)	P value
ABO compatibility				
Match	1.0		1.0	
Major mismatch	1.24 (0.77-1.99)	0.38	0.84 (0.42-1.67)	0.61
Minor mismatch	2.29 (1.47-3.55)	<0.0001	1.78 (0.98-3.26)	0.59
Bidirectional	1.37 (0.65-2.88)	0.41	0.87 (0.26-2.93)	0.82
ATG				
Yes	1.0		1.0	
No	0.81 (0.68-3.08)	0.63	0.31 (0.03-3.04)	0.31
Primary Disorder				
Benign	1.0		1.0	
Malignant	2.62 (1.66-4.14)	<0.0001	20.5 (6.3-66.9)	<0.0001
Recipient Age	1.007 (0.988-1.027)	0.45	1.001 (0.975-1.027)	0.96
Donor Age	0.998 (0.979-1.017)	0.81	0.971 (0.953-0.989)	0.002
AGVHD Grade ≥ II	0.40 (0.14-1.12)	0.08	0.77 (0.14-4.21)	0.76
Skin	0.63 (0.18-2.19)	0.47	N/A	
GI	1.80 (1.26-2.57)	0.001	1.52 (0.28-8.11)	0.62
Liver	2.70 (1.18-6.17)	0.018	0.78 (0.16-3.83)	0.76
Chronic GVHD	0.77 (0.48-1.25)	0.29	0.53 (0.31-0.89)	0.02
No	1.0		1.0	
Limited	0.48 (0.29-0.81)	0.006	0.73 (0.40-1.33)	0.30
Extensive	0.94 (0.60-1.47)	0.79	0.23 (0.09-0.63)	0.004
CD34 Cell dose	1.010 (0.925-1.103)	0.82	1.123 (0.995-1.267)	0.06
Conditioning Regimen				
Non-MA	1.0		1.0	
MA	1.67 (0.85-3.26)	0.13	2.41 (0.74-7.87)	0.14
Gender Mismatch				
D-R Sex-Match	1.0		1.0	
Female to Male	1.42 (0.93-2.19)	0.10	1.30 (0.72-2.34)	0.37
Male to Female	1.13 (0.70-1.79)	0.61	0.84 (0.44-1.59)	0.59
HLA Matching				
Sibling	1.0			
Match other relative	2.05 (0.64-6.59)	0.23	N/A	
Mismatch relative	1.46 (0.31-6.74)	0.62	8.12 (0.84-78.4)	0.07
ANC recovery	1.030 (0.983-1.079)	0.21	1.057 (0.95-1.175)	0.31
Plt recovery	0.995 (0.964-1.026)	0.73	0.985 (0.938-1.033)	0.52

Table 3 also includes Cox regression multivariate analysis results for RFS. ABO incompatibility was not correlated with RFS (Figure 1-B). Malignant disease decreased RFS (RR: 20.5, CI: 6.3-66.9, p-value<0.0001). In contrast,donor’s age and extensive cGVHD improved RFS (RR: 0.971, CI: 0.953-0.989, p- value=0.002 and RR: 0.23, CI: 0.09-0.63, p- value=0.004, respectively).

**Figure1-B:**
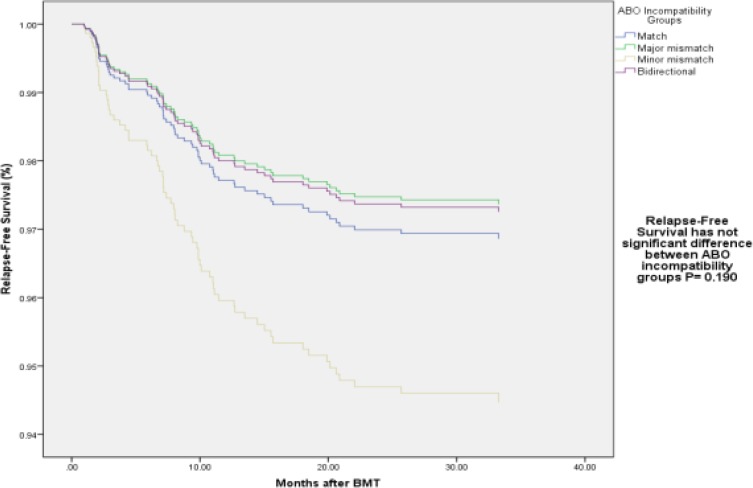
Relapse-Free Survival by ABO incompatibility Groups in multivariate Cox regression analysis

The cumulative incidence of relapse was not significantly different among the four groups (Figure1-C).Malignant primary disorder increased relapse rate(RR:31.39, CI: 8.33-118.26,p-value<0.0001). 

**Figure1-C:**
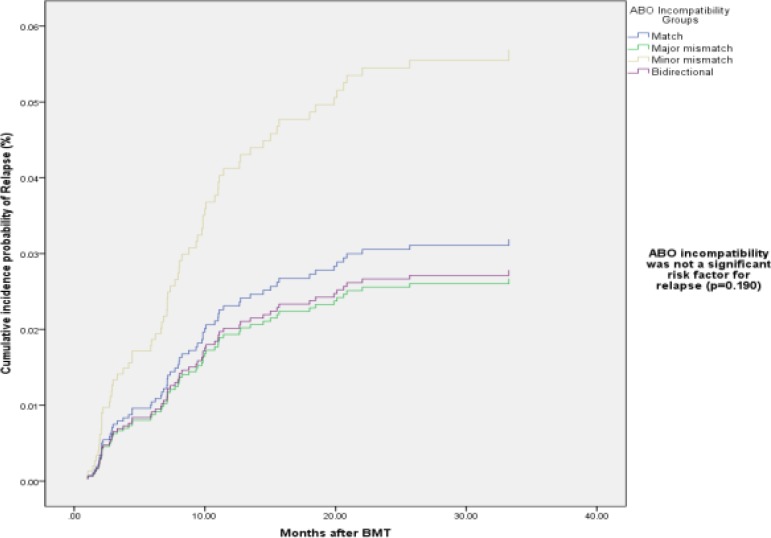
Cumulative incidence probability of Relapse by ABO incompatibility groups

Extensive cGVHD and donor’s age decreased relapse rate(RR: 0.24, CI: 0.09-0.65,p-value=0.005 and RR:0.970, CI: 0.950-0.991, p-value=0.005, respectively).Donation from a mismatched relative was a risk factor for relapse (RR: 12.39, CI: 1.10-140.32, p-value=0.04) (Table4).

**Table 4:** multivariate analysis for Relapse and Non-Relapse Mortality

Factors	Relapse				Non-Relapse Mortality			
	Relative Risk (95% CI)		P value		Relative Risk (95% CI)		P value	
ABO compatibility								
Match	1.0				1.0			
Major mismatch	0.71 (0.31-1.60)		0.41		1.18 (0.52-2.65)		0.70	
Minor mismatch	1.47 (0.67-3.22)		0.33		1.65 (0.71-3.84)		0.24	
Bidirectional	0.80 (0.20-3.20)		0.75		1.89 (0.56-6.43)		0.31	
ATG								
Yes	1.0				1.0			
No	0.24 (0.02-3.60)		0.30		0.86 (0.29-2.52)		0.78	
Primary Disorder								
Benign	1.0				1.0			
Malignant	31.39 (8.33-118.26)		<0.0001		1.04 (0.33-3.29)		0.95	
Recipient Age	1.001 (0.970-1.033)		0.96		1.019 (0.986-1.053)		0.26	
Donor Age	0.970 (0.950-0.991)		0.005		1.024 (1.005-1.044)		0.02	
AGVHD Grade ≥ II	0.68 (0.11-4.33)		0.69		0.25 (0.04-1.66)		0.15	
Skin	N/A				1.72 (0.30-9.76)		0.54	
GI	1.73 (0.28-10.59)		0.56		3.32 (1.84-6.00)		<0.0001	
Liver	0.55 (0.09-3.28)		0.51		1.86 (0.50-6.98)		0.36	
Chronic GVHD	0.21 (0.07-0.62)		0.004		2.26 (1.11-4.60)		0.02	
No	1.0				1.0			
Limited	0.80 (0.40-1.60)		0.52		0.40 (0.16-1.01)		0.05	
Extensive	0.24 (0.09-0.65)		0.005		2.49 (1.31-4.72)		0.005	
CD34 Cell dose	1.12 (0.98-1.30)		0.11		0.97 (0.83-1.14)		0.72	
Conditioning Regimen								
Non-MA	1.0				1.0			
MA	2.70 (0.77-9.47)		0.12		1.31 (0.50-3.44)		0.59	
Gender Mismatch								
D-R Sex-Match	1.0				1.0			
Female to Male	1.36 (0.68-2.74)		0.39		1.81 (0.86-3.82)		0.12	
Male to Female	0.79 (0.37-1.68)		0.54		1.63 (0.75-3.54)		0.22	
HLA Matching							
Sibling	1.0			1.0			
Match other relative	N/A			2.90 (0.52-16.18)		0.23	
Mismatch relative	12.39 (1.10-140.32)	0.04		0.73 (0.6-8.25)		0.80	
ANC recovery	1.02 (0.90-1.16)	0.71		1.02 (0.94-1.11)		0.61	
Plt recovery	0.97 (0.92-10.3)	0.38		0.99 (0.93-1.05)		0.68	

Multivariate analysis of NRM revealed nodifference among ABO incompatibility groups .Donor’s age weakly increased NRM rate (RR: 1.024, CI: 1.005-1.044, p-value=0.02). Acute GI, GVHD and cGVHD were risk factors for increasing NRM rate (RR: 3.32, CI: 1.84-6.00, p-value<0.0001 and RR: 2.26, CI: 1.11-4.60, p-value=0.02, respectively). While extensive cGVHD significantly increased NRM (RR: 2.49, CI: 1.31-4.72, p-value=0.005), limited cGVHD decreased that rate, but it was not significant (RR: 0.40, CI: 0.16-1.01, p-value=0.05) (Table 4, Figure1-D).

**Figure1-D:**
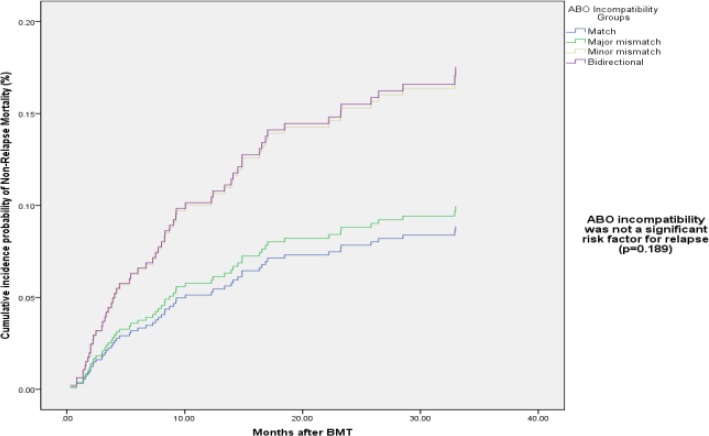
Cumulative incidence probability of NRM by ABO incompatibility groups

Multivariate analysis of acute and chronic GVHD showed that MA regimen increased aGVHD(RR: 1.81, CI: 1.003-3.27, p-value=0.049).Omitting ATG (Antithymocyte) from conditioning regimen increased cGVHD (RR: 2.28, CI: 1.35-3.83, p- value=0.002). Both donor’s age and aGVHDgrade≥II were risk factors for cGVHD(RR: 1.014, CI: 1.001-1.027, p-value=0.04 and RR: 1.49, CI: 1.02-2.20, p-value=0.04) and also female to male donation increased cGVHD(RR: 2.01, CI: 1.28-3.16, p-value=0.002)

Pure red cell aplasia (PRCA) occurred in two male patients (with ALL and SAA) in major mismatched group; one of whom had received cells from female and the other from male HLA - identical sibling donor.They received MA and non-MA regimen as conditioning, respectively.aGVHDof liver (grade II-III) and also cGVHD were presented in both patients.

## Discussion

 Approximately one-third of bone marrow or peripheral blood stem cell transplantations are performed with ABO blood group incompatibility.^2^^,^^13^

In this study, we evaluated the impact of ABO mismatch on outcomes such as OS, RFS, and NRM, time to engraftment, relapse and also GVHD**.** A decrease in OS was only observed in patients undergoing minor blood group mismatched HCT.

We observed that a decrease in OS occurred only in patients with minor blood group mismatched transplantations. RFS was lower in minor mismatched grafts, but the difference was not statistically significant. Similar to our results, Ozkurt et al.’s and Logan et al.’s studies also reported a significantly shorter OS in recipients with minor ABO-mismatched grafts.^14^^,^^15^Stussi et al. observed an independent decrease in survival after bidirectional ABO-incompatible SCT, but it was not found in the minor or major ABO-incompatible groups.^2^Three hundred thirty-eight patients with ABO-incompatible SCT were evaluated by Mielcarek et al., and there were no significant differences in survival and GVHD among the ABO-incompatible groups.^3^Some other studies have also reported no relationship between ABO groups and OS.^3^^,^^5^^,^^6^^,^^16^In a large retrospective study conducted in Japan, OS was significantly lower in major and minor mismatched groups than the AB0-identical group.^17^Time to ANC engraftment was not different among study groups. This result was confirmed in Mielcarek et al.’s and Kim et al.’sstudies.^3^^,^^6^We observed significant difference in mean platelet engraftment time among four groups and major mismatched group showed the maximum platelet engraftment time. Japanese study showed engraftment delay in neutrophils, platelets, and erythrocytes in transplants with major incompatibility.^17^

Minor and bidirectional mismatched groups required more packed cell infusion than matched group. Major mismatches received more platelet infusion than others. Although Ozkurt et al. and Kim et al. studies have shown that ABO-mismatched groups had no greater transfusion requirements than ABO-identical ones,^6^^,^^14^ some other studies have shown that ABO-incompatible group has greater transfusion requirements.^3^^,^^5^

Similar to Seebach et al. study,^16^ we observed no difference in relapse and NRM between ABO-pairs. But Kimura et al. showed higher NRM in the major and minor mismatched groups. Meanwhile, just like our results,they did not find any significant difference in rate of relapse.^16^In 2015, Biology of Blood and Marrow Transplantation Journal published an article reporting an increase in NRM of minor mismatched groups.^15^

Some studies that ABO incompatibility may be associated with increased risk of GVHD.^17^^-^^19^ In one report, minor ABO incompatibility was related with a higher risk of severe acute GVHD in comparison to other groups,^20^ but in our study, aGVHD and cGVHD were not statistically correlated with ABO compatibility. Mielcarek et al. and also Kim et al. have also reported similar results.^3^^,^^6^

Pure red cell aplasia (PRCA) occurred in two male patients (with ALL and SAA) of our major mismatched group. They received transplantation from female and male HLA - identical sibling donors, respectively. PRCA has also been reported after major ABO-incompatible stem cell transplantation in other studies.^2^^, ^^21^^, ^^22^ Finally, in our study, multivariate analysis revealed that MA regimen increased aGVHD and omitting ATG, donor’sage,aGVHD≥II and female to male donation were all risk factors for developing cGVHD.

Since unfavorable outcomes and complications such as decrease in OS, the need for more transfusion and PRCA are statistically significant in ABO incompatible SCT, we suggest that in clinical practice, if a given patient has several suitable donors, the one with a compatible ABO blood group would improve outcomes of the transplantation.

Considering relatively diverse results on this topic in the literature, a study with a larger sample size and also a meta-analysis could probably help achieve more accurate results.

## CONCLUSION

 Since unfavorable outcomes such as decrease in OS and the need for more transfusions are statistically significant in ABO incompatible SCT and also complicationsuch as PRCA is observed in these patients, we suggest that in clinical practice, if a given patient has several suitable donors, the one with a compatible ABO blood group would improve outcomes of the transplantation.

Considering relatively diverse results on this topic in the literature, a study with a larger sample size and also a meta-analysis could probably help achieve more accurate results.
